# Heparin-Induced Thrombocytopenia under Mechanical Circulatory Support by Large Impella for Acute Cardiogenic Shock

**DOI:** 10.3390/jcdd8120161

**Published:** 2021-11-25

**Authors:** Yukiharu Sugimura, Sebastian Bauer, Moritz Benjamin Immohr, Derik Franz Hermsen, Ralf Westenfeld, Udo Boeken, Hug Aubin, Igor Tudorache, Artur Lichtenberg, Payam Akhyari

**Affiliations:** 1 Department of Cardiac Surgery and Research Group for Experimental Surgery, Medical Faculty and University Hospital Düsseldorf, Heinrich Heine University Hospital, 40225 Düsseldorf, Germany; Yukiharu.Sugimura@med.uni-duesseldorf.de (Y.S.); Sebastian.Bauer@med.uni-duesseldorf.de (S.B.); Moritz.Immohr@med.uni-duesseldorf.de (M.B.I.); Udo.Boeken@med.uni-duesseldorf.de (U.B.); Hug.Aubin@med.uni-duesseldorf.de (H.A.); igor.tudorache@med.uni-duesseldorf.de (I.T.); payam.akhyari@med.uni-duesseldorf.de (P.A.); 2 Institute of Clinical Chemistry and Laboratory Diagnostics, Medical Faculty and University Hospital Düsseldorf, Heinrich Heine University Hospital, 40225 Düsseldorf, Germany; Hermsen@med.uni-duesseldorf.de; 3 Department of Cardiology, Angiology and Pulmonology, Medical Faculty and University Hospital Düsseldorf, Heinrich Heine University Hospital, 40225 Düsseldorf, Germany; Ralf.Westenfeld@med.uni-duesseldorf.de

**Keywords:** cardiogenic shock, Impella, heparin-induced thrombocytopenia, anticoagulation

## Abstract

Despite the critical feature of heparin-induced thrombocytopenia (HIT) for patients on mechanical circulatory support, reports on its incidence and outcome are still scarce. Thus, we report on clinical features of HIT in patients under Impella 5.0 or 5.5 (Abiomed Inc., Danvers, MA, USA) (Impella 5+) support for acute cardiogenic shock (CS) by focusing on observed thrombotic events. Between November 2018 and December 2020, a total of 56 consecutive patients were enrolled in a single-center retrospective study. A total of 21 patients (37.5%) were tested for HIT, and 6 (10.7%) proved positive for HIT at 10.5 ± 2.89 days after the first heparin administration during current admission. Interestingly, thrombocyte counts dropped under Impella support in all groups (all cases, no HIT test, and HIT negative group: *p* < 0.001, HIT-positive group: *p* = 0.001). All HIT-positive patients were switched from heparin to argatroban. HIT-associated thrombotic events were observed in two cases resulting in Impella dysfunction due to pump thrombosis (*n* = 1) and left ventricular (LV) thrombus formation (*n* = 1). Under large Impella support, the prevalence of HIT was relatively high. Further, thrombocytopenia does not deliver a high specificity in the setting of Impella 5+ support. Considering HIT manifestation, a routine HIT test may be considered to avoid critical thrombotic adverse events.

## 1. Introduction

The use of an extracorporeal circulatory system, e.g., veno-arterial extracorporeal membrane oxygenation therapy (va-ECMO), is widely adopted in many countries as a solid and reliable tool for mechanical circulatory support (MCS) in patients with severe heart failure (HF) or cardiogenic shock (CS). In this patient cohort, anticoagulation is indispensable for preventing thrombotic adverse events. However, bleeding disorders, as well as thromboembolic events, are considered as relevant complications of patients under MCS by va-ECMO. 

Recently, microaxial pumps, i.e., Impella (Abiomed Inc., Danvers, MA, USA), have emerged as an alternative tool for temporary MCS. Despite the substantially different mode of action of Impella omitting extracorporeal blood circulation and the need for an oxygenator, MCS by Impella yet requires anticoagulation. As recommended by the manufacturer, a purge fluid containing anticoagulant is constantly administered to wash the Impella pump and prevent patient blood from entering the motor. In such a setting, anticoagulation management is crucial to avoid pump thrombosis, and unfractionated heparin (UFH) is utilized as the first choice for anticoagulation.

However, heparin-induced thrombocytopenia (HIT) is a well-described and severe complication in patients with continuous intravenous heparin administration. Antibodies to the platelet factor 4 and heparin complex play a central role in HIT development, resulting in a fatal hypercoagulation state without causing bleeding [1]. In front of this background, HIT development may cause life-threatening conditions in CS patients under Impella support, as the risk of pump dysfunction or thromboembolic events due to HIT-induced hypercoagulation is considerably increased. 

Even though HIT is a crucial issue in utilizing the Impella system, only a few cases are reported in the literature [2,3,4,5,6,7]. Further, reports are even more limited in the setting of large surgical Impella systems, i.e., Impella 5 or 5.5 (Impella 5+).

Thus, in this report, we documented our clinical experiences of Impella 5+ and analyzed HIT-positive patients to review the strategy for early HIT diagnosis and early modification of Impella management.

## 2. Materials and Methods

### 2.1. Study Design and Data Collection

**Figure 1:**
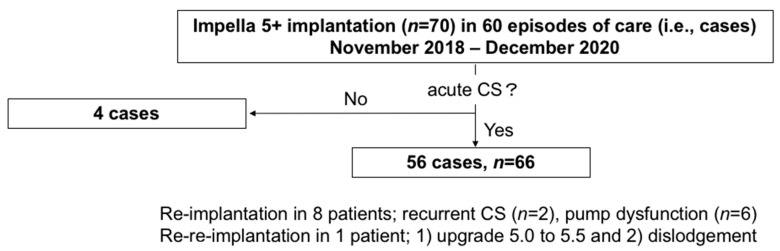
The graphic explanation of the study population. 66 applications in 56 episodes of care (i.e., cases), arising from 70 Impella 5+ (5.0 or 5.5) implantation in 60 cases. CS, cardiogenic shock; Impella 5+, Impella 5.0 or 5.5.

This study was designed to determine the incidence of HIT in patients with acute CS receiving Impella 5+ and evaluate patient outcomes depending on the presence of HIT. Between November 2018 and December 2020, we performed a total of 70 consecutive Impella 5+ (*n* = 67 for Impella 5.0; *n* = 3 for Impella 5.5) implantations in 60 episodes of care (i.e., cases) in our institution. Four patients who underwent off-pump coronary artery bypass grafting under Impella 5.0 support in the setting of ischemic cardiomyopathy without CS were excluded. Thus, a total of 56 acute CS cases were enrolled in this study (Figure 1). Among these 56 cases, 8 patients received two Impella 5+ systems, 2 of whom underwent Impella 5+ re-implantation due to recurrent CS. The remaining 6 patients underwent Impella 5+ exchange due to pump dysfunction. Finally, another patient underwent three Impella 5+ implantation because of (1) upgrade 5.0 to 5.5 and (2) dislodgement of the second Impella. 

All data were collected retrospectively from our medical archive systems. Analyzed data included preoperative characteristics and information about the postoperative clinical course.

### 2.2. Standard Anticoagulation Management and HIT Diagnosis under Impella 5+

The anticoagulation for Impella 5+ was managed according to the current recommendation of the manufacturer. As far as anticoagulation for purge solution, 5% dextrose in water (D5W) with UFH (50 U/mL) was used as standard. Moreover, systemic UFH was also administrated to achieve target activated partial thromboplastin time (aPTT) above 45 s and below 72 s. aPTT was monitored every 8 h until aPTT values became stable, from there on in 24-h intervals. However, in patients with clinical bleeding tendency, i.e., coagulopathy, or in postoperative cases, anticoagulation was adopted to the clinical situation in a case-by-case manner, in the extreme scenario of intractable postoperative bleeding also considering temporary pause of anticoagulation therapy. In such a case, we restarted the anticoagulation once the bleeding situation had improved. 

HIT test was performed in suspected cases with a marked drop in platelet counts. In general, some scoring systems, e.g., the 4Ts scoring system [8] or the HIT expert probability (HEP) score [9], were performed followed by blood test. However, because of unmatched scoring criteria for CS patients supported by Impella 5+, these scoring systems were not adopted into clinical routine at our institution. HIT diagnostic was performed using enzyme-linked immunosorbent assay (ELISA) (LIFECODES PF4 enhanced assay; Immucor GTI Diagnostics, Waukesha, WI, USA) with optical density (OD) measurements, followed by a high-dose heparin confirmatory ELISA when first ELISA was positive (OD > 0.4) and heparin-induced platelet aggregation test (HIPA) and HIT-IgG specific antibodies measurements (OD) in case of conflicting results between first ELISA and a high-dose heparin confirmatory ELISA. According to our institutional protocol, patients are considered as HIT positive (1) if first ELISA and a high-dose heparin confirmatory ELISA were positive, and (2) if first ELISA was positive with strongly positive result for HIT-IgG specific antibodies (OD > 1), or (3) HIPA was positive. Highly positive results for HIT-IgG are regarded as diagnosing HIT-positive status by the clinical decision because thrombosis will likely happen and anticoagulation regimen, i.e., heparin administration, must be changed to other, non-heparin-based schemes, e.g., argatroban, irrespective of the result of HIPA test.

In the setting of HIT, argatroban (2 μg/kg/min) was initially administrated intravenously and modified according to aPTT for systemic anticoagulation. As far as the addition of anticoagulation agents to purge solution is concerned, no proper guidelines existed at the time of the reported cases. Likewise, a case-by-case decision was made in an interdisciplinary team consisting of a cardiac surgeon, an intensive care specialist, an haemostaseology specialist, and in compliance with the recommendations of the clinical specialists of the manufacturer.

### 2.3. Statistical Analysis

The statistical analysis was conducted in a patient-based manner in this study. All analyses were performed with the Statistical Package for Social Sciences^®^ (SPSS) 25.0 (IBM, Chicago, IL, USA). Using this program, descriptive and comparative statistics were performed. Unless otherwise indicated, continuous variables were compared using the Mann–Whitney U test for two groups’ analysis. For three group analysis, the Kruskal–Wallis test was conducted. A Chi-Quadrat-Test was conducted for nominal scaled variables. However, Fisher’s exact test was adapted instead of the Chi-Quadrat-Test for a minimum expected value of less than 5. *p*-values less than 0.05 were considered statistically significant. 

## 3. Results

### 3.1. Baseline Clinical Characteristics

Among 56 patients, we performed HIT tests in a total of 21 patients (37.5%), 6 of whom (28.6% of 21 patients with HIT test, 10.7% of the entire cohort) were confirmed to be HIT positive. For the descriptive analysis, we assigned all patients in three groups; no HIT test group (*n* = 35, 62.5%), HIT-positive group (*n* = 6, 10.7%), and HIT negative group (*n* = 15, 26.8%) (Table 1). Most of all, preoperative clinical parameters represented no significant difference between groups. However, there was a larger proportion of patients with acute coronary syndrome/ischemic cardiomyopathy in no HIT test group as an underlying cause for CS (*p* < 0.05). Further, the C-reactive protein value was also statistically higher in the no HIT test group than the HIT negative group (<0.05). As far as the in-hospital mortality is concerned, 33 patients of the total cohort (57.9%) did not survive, whereas, in HIT-positive group, 2 patients (33.3%) died, which suggested no statistically significant difference.

### 3.2. HIT-Associated Clinical Features and Outcomes

As one of the HIT-associated manifestations, we examined circulating thrombocyte counts during the clinical episode in HIT-positive group. Table 2 demonstrates maximum and minimum thrombocyte counts until the confirmation of the diagnosis of HIT. In all HIT cases, circulating thrombocytes were reduced by more than 50%, on average by 76.1%. HIT was diagnosed at 10.5 ± 2.89 days after the first heparin administration during the current admission. On the other hand, the HIT test was performed in the HIT negative group at 8.00 ± 4.90 days (*p* = 0.15). In each case, the first heparin administration was in the context of either veno-arterial extracorporeal membrane oxygenation (va-ECMO) implantation, open-heart surgery, or Impella implantation, with no statistical difference between the study sub-cohorts (i.e., HIT-positive or negative). HIT-associated thrombotic events under Impella 5+ support were observed in two cases, presenting as Impella dysfunction due to pump thrombosis (*n* = 1) and as left ventricular (LV) thrombus formation (*n* = 1) (Table 3).

### 3.3. Changes in Platelet Count during Impella Support

**Figure 2:**
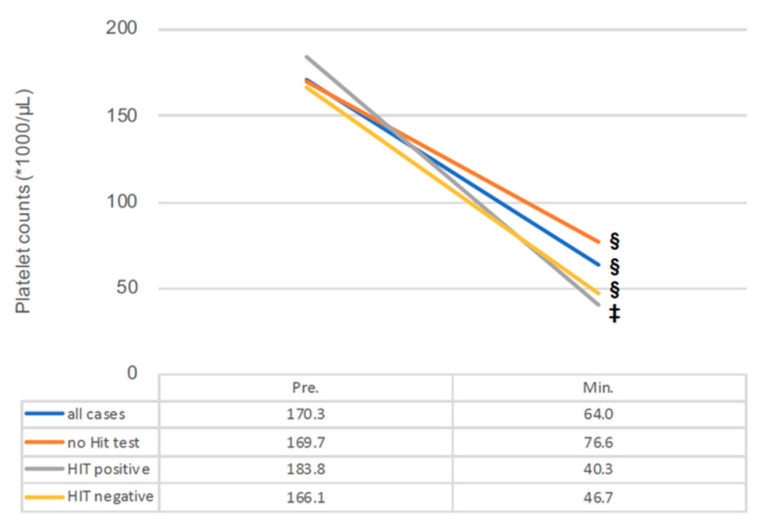
Changes in platelet counts under Impella 5+ support with or without clinically triggered HIT diagnostic. Min., minimum; HIT, heparin-induced thrombocytopenia; Impella 5+, Impella 5.0 or 5.5; Pre., preimplantation of Impella 5+; ‡, *p* < 0.005; §, *p* < 0.001. *, multiplication.

The survey of platelet counts using Impella 5+ depending on HIT diagnostic is shown in Figure 2. Interestingly, all groups demonstrated statistically dominant thrombocyte drops (all cases, no HIT test and HIT negative group: *p* < 0.001, HIT-positive group: *p* = 0.001).

### 3.4. Outcome of Survivors after Positive HIT Diagnostic

**Figure 3:**
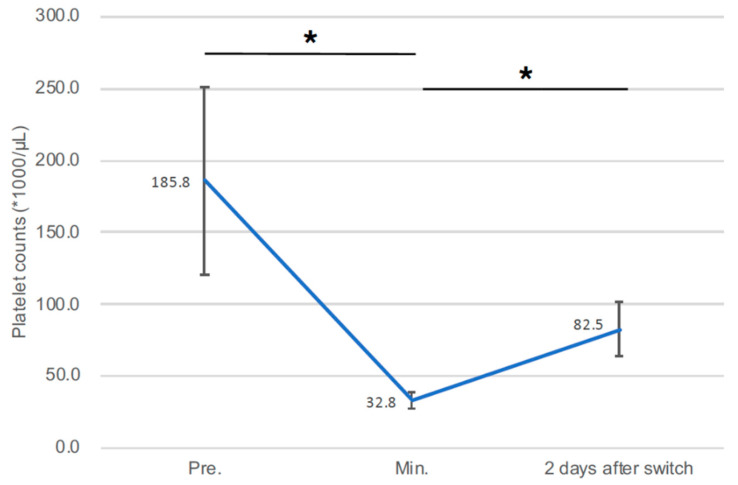
The drop and recovery of platelet counts in four survivor patients under Impella 5+ support accompanied by HIT-positive diagnostic. Min., minimum; HIT, heparin-induced thrombocytopenia; Impella 5+, Impella 5.0 or 5.5; Pre., preimplantation of Impella 5+; * *p* < 0.05.

The clinical outcomes of four survivors after HIT-positive diagnostic are presented in Table 4. Argatroban was used for purge solution and systemic anticoagulation. No patient suffered from neurological deficits. All four patients were discharged with an average of 47.3 ± 15.6 days. Thrombocyte counts significantly improved after the conversion of anticoagulation therapy to Argatroban (platelets count (* 1000 μL) at a minimum level during Impella use vs. 2 days after conversion: 32.8 ± 5.56 vs. 82.5 ± 18.6, *p* = 0.02) (Figure 3).

### 3.5. Impella Dysfunction Due to Pump Thrombosis Associated with HIT

The clinical course of a patient with pump thrombosis associated with HIT is shown in Table S1. A 62-year-old man who suffered from giant-cell myocarditis was referred to us in CS status with cardiac index 1.1 L/min/m^2^ as determined by right-heart catheterization and LV ejection fraction (EF) of 24% as determined by echocardiography. Due to fulminant myocarditis with high catecholamine levels, va-ECMO became necessary, immediately followed by axillary Impella 5.0 implantation on admission day. A HIT test was repeatedly performed because of a decrease in circulating thrombocyte counts. However, the HIT test initially proved to be negative, although OD became tendentially higher. Thus, according to the standard protocol, UFH was further applied for systemic anticoagulation and supplemented to the purge solution. On the eighth postoperative day (POD), repeated HIT tests resulted positive, so anticoagulation therapy was switched from UFH to argatroban. On the ninth POD, purge flow rate decreased, and Impella flow also dropped to levels below 2 L/min despite choosing P7 Impella setting. Finally, Impella flow decreased to less than 1 L/min, and purge pressure increased to over 1000 mmHg. In an analysis of all available parameters and compliance with recommendations given by clinical specialists of the manufacturer, system dysfunction due to pump thrombosis was diagnosed. Therefore, we explanted the dysfunctional Impella 5.0 on the 10th POD. Fortunately, LV function had meanwhile markedly improved so that the patient did not require a continuation of MCS. Transthoracic echocardiography (TTE) revealed 47% LVEF after va-ECMO explantation on the 18th POD. After a steady recovery course, the patient was finally on the 27th POD transferred to a peripheral hospital in a satisfying condition for further re-convalescence.

### 3.6. LV Thrombus Associated with HIT under Impella 5.0 Support

The clinical course of another patient with LV thrombus associated with HIT is shown in Table S2. The 79-year-old male patient underwent minimally invasive mitral valve replacement due to severe mitral regurgitation and a massive calcified posterior mitral leaflet. Perioperatively, LV myocardial rupture was observed, which required LV repair after conversion to sternotomy, followed by va-ECMO implantation due to minimal LV contraction and systemic inflammatory activation. In the initial postoperative course, stabilization was achieved without other assist devices, while the ejection of LV under va-ECMO and catecholamine therapy was preserved. However, gradual pulmonary congestion prompted us to escalate the MCS strategy with the addition of LV unloading. Impella 5.0 was implanted via the right subclavian artery on the 13th POD. One day after, routine laboratory testing demonstrated higher D-dimers. Thus, HIT test was performed and resulted positive. During the afternoon on the same day Impella flow markedly decreased despite a high Impella setting. TTE indicated a large LV thrombus despite apparently optimal anticoagulation according to PTT values. In an interdisciplinary conference reviewing clinical and laboratory aspects, therapy withdrawal was determined in front of emerging multiple organ failure and evident LV-thrombosis in the 15th POD.

## 4. Discussion

The main results of this study are: (1) the incidence of HIT under Impella 5+ lies at 10.7% and is diagnosed at 10.5 ± 2.89 days after initial heparin exposure; (2) a marked drop in thrombocyte counts was present in all Impella patients. However, with the most substantial relative drop observed in HIT-positive patients (average 76.1%), and (3) in the small sub-cohort of Impella patients developing HIT a high rate of 33.3% was observed for thrombotic clinical events.

### 4.1. Diagnostic of HIT with Impella as a Part of MCS

Generally, the prevalence of HIT in critically ill patients is reported to range under 1%, whereas the incidence of HIT post cardiovascular surgery is reported at 1–2% [10]. This study indicated a distinctly higher prevalence of HIT (10.7%) in patients with acute CS under Impella 5+ support. Current therapy schemes for MCS of the cardiopulmonary system are strongly related to heparin administration. Moreover, most patients who require large Impella due to CS typically suffer from critical illness, necessitating interventional therapies or open-heart surgical procedures. Further, such CS patients likely need resuscitation with or without temporary MCS, i.e., va-ECMO as the first treatment modality. Operative or interventional therapy or further MCS escalation often follows after very initial stabilization. In this sense, especially patients under large Impella support carry a medical history with prior heparin administration, e.g., UFH. We think that the latter factor is one of the contributors to the higher rate of HIT incidence. In this study, the incidence of HIT is almost the same as the past studies on HIT under MCS [11]. Regarding the onset time of HIT, our data suggest that HIT becomes evident at 10.5 ± 2.89 days after the first heparin administration since a current hospital admission, which is almost the exact timing of HIT according to previous studies (5–10 days) [1]. 

We performed HIT tests in one-third of our Impella 5+ patients (21/56), who had relevant thrombocytopenia suspicious for HIT. An early HIT diagnosis is indispensable for favorable therapy outcomes because of its crucial life-threatening condition, i.e., hypercoagulation. There may not be a constant high level of attention present in routine clinical practice towards the potential HIT development. Occasionally, due to the absence of suspicion about HIT, HIT seems to remain “hidden.” In other words, HIT positive cases might probably be just “the tip of the iceberg.” This puts a timely and efficient diagnosis finding process into the center of therapy efforts, warranting a constantly high awareness for HIT, especially in patients with temporary MCS. For the proper differential HIT diagnosis, the 4Ts scoring system (thrombocytopenia, timing of platelet decreases, thrombosis or other sequelae, and other causes of thrombocytopenia) was established [8]. In a previous meta-analysis, a cut-off value of ≥4 in the 4Ts scoring system was shown to result in 99% sensitivity and 54% specificity for HIT [12]. The report suggests the modification of anticoagulants, e.g., discontinue UFH and start an alternative anticoagulant in the case of cut-off ≥4, no matter whether HIT test proved positive or not. This strategy may be adopted for patients with acute CS under Impella. However, in our opinion, more evidence is needed to demonstrate the validity of the 4Ts scoring system as appropriate in the setting of Impella support. For example, looking at scoring categories of 4Ts, the category “timing (of platelet count fall)” is scored from >5 days after first heparin administration or <1 day. In fact, thrombocytes in patients who have received MCS automatically dropped after MCS begins because of the share stress of pumps (fragmentation), but also due to other events, such as biointerface activation (e.g., in the case of va-ECMO). Moreover, some patients receive heparin exposure with more than one day but less than 5 day intervals until implementation of MCS, which results in an artefact to the typical time windows described in 4Ts scoring system. Further, thinking about patients’ baseline characteristics, all patients suffered from acute CS, some patients have been resuscitated, some patients undergo va-ECMO implantation, whereas some other patients are postoperative patients. In this situation, the scoring of the category “other causes for thrombocytopenia” seems to be difficult. HEP score has also similar problems, although here at least the contribution of va-ECMO and other MCS components to platelet drops are acknowledged with negative points. Moreover, regarding the scoring of the category “thrombosis or other sequelae,” once thrombotic manifestations appear under temporary MCS, it may be already too late. 

Arising from this dilemma, we conducted this retrospective observational study to examine HIT incidence, to determine how HIT may impact on clinical outcomes, and to elucidate the clue for HIT strategy in CS patients with Impella 5+. Then we observed that the decrease in thrombocytes was manifested in the HIT-positive group and in all other groups. Thus, we suggest that the HIT test be performed routinely, and anticoagulation management be modified if the thrombocytes are reduced, until HIT diagnosis is validated.

Regarding risk factors of HIT, no risk factors were determined in this study, although female gender and major surgery were reported as risk factors in previous reports [13,14]. This finding may be explained by the fact that CS status is a distinct situation from the background of most previous studies. The previous reports associated with HIT may not be easily adapted to the specific setting in patients with acute CS under temporary MCS support, e.g., Impella.

### 4.2. Anticoagulation in HIT with Impella as a Part of MCS

Concerning anticoagulation in HIT, anti-thrombin inhibitors, e.g., argatroban [3,6,7] or bivalirudin [2,4,5], are preferably administrated for systemic anticoagulation. Hence, anti-thrombin inhibitors are occasionally used as an additive agent in purge solutions. However, the use of alternative anticoagulant-containing purge solutions in patients requiring Impella has not yet been thoroughly discussed. Notably, Syzmanski et al. reported the experience of bivalirudin instead of UFH for systemic anticoagulation and purge solution. They determined bivalirudin doses for purge solution depending on the required amounts for systemic anticoagulation (target aPTT: 60 s–80 s). According to the last report, half of the dose needed for systemic anticoagulation was administrated in purge solution (D5W), whereas systemic bivalirudin doses were subtracted by the respective amount [2].

On the other hand, another report documents the successful approach with no anticoagulant in purge solution under therapeutic systemic anticoagulation with bivalirudin [4]. In the case of argatroban, anticoagulation monotherapy with a purge solution containing a concentration of 0.1 mg/mL argatroban has been reported [7]. Reviewing the latter concepts, although there is still no consensus about the ideal anticoagulation regimen for Impella in the case of HIT, we should not forget the pharmacokinetics of the two most widely used anti-thrombin inhibitors; argatroban is metabolized in the liver, whereas bivalirudin is metabolized in the kidney. Depending on the patients’ status, we must pay attention to the doses of both anticoagulants; otherwise, coagulopathy problems may likely happen.

There are several limitations to this study. First, this manuscript deals with a retrospective observational study on a limited cohort size of non-randomized patients at a single center. Potential systematic measurement errors and also unidentified bias can affect the outcomes. Secondly, our analysis does not cover data on long-term outcomes. Third, regarding HIT diagnostics, we additionally performed a HIPA test only in the case of positive first ELISA and negative high-dose heparin confirmatory ELISA. For platelet function, the HIPA test should be performed to confirm if HIT-antibody occurred thrombocytes aggregation. Further studies are warranted for confirmation of our therapy strategy in HIT under Impella.

## 5. Conclusions

Under large Impella support, the prevalence of HIT was relatively high (10.7%), with the onset time at 10.5 ± 2.89 days after the first heparin administration during current admission. 33.3% of all HIT patients suffered from critical hypercoagulation events. In the light of these findings, routine HIT tests and a liberal change of anticoagulation regimen upon the drop of thrombocyte counts appear favorable for patients under Impella support. Anti-thrombin inhibitors, argatroban, or bivalirudin will be an alternative anticoagulant instead of UFH. However, ideal dosage and administration pathways remain the subject of future studies. Further, the findings of this study are hypothesis-generating, which need to be confirmed in future studies involving larger patient cohorts, ideally in the setting of a randomized controlled trial.

## Figures and Tables

**Table 1:** Baseline clinical characteristics and mortality.

	All Cases (*n* = 56)	No HIT Test (*n* = 35)	HIT Positive (*n* = 6)	HIT Negative (*n* = 15)	*p*
Age (year)	61.8 ± 11.6	63.1 ± 12.2	57.0 ± 15.1	60.7 ± 8.17	ns
Male, *n* (%)	47 (83.9)	29 (82.9)	5 (83.3)	13 (86.7)	ns
INTERMACS profiles I, *n* (%)	25 (44.6)	16 (45.7)	1 (16.7)	8 (53.3)	ns
Arterial hypertension, *n* (%)	33 (58.9)	23 (65.7)	4 (66.7)	6 (40.0)	ns
Hyperlipidemia, *n* (%)	14 (25.0)	10 (28.6)	2 (33.3)	2 (13.3)	ns
Diabetes, *n* (%)	21 (37.5)	14 (40.0)	1 (16.7)	6 (40.0)	ns
Peripheral vascular disease, *n* (%)	6 (10.7)	3 (8.6)	0 (0.0)	3 (20.0)	ns
Arrhythmia, *n* (%)	19 (33.9)	11 (31.4)	0 (0.0)	8 (53.3)	ns
COPD, *n* (%)	3 (5.4)	2 (5.7)	0 (0.0)	1 (6.7)	ns
Nicotine abuses, *n* (%)	16 (28.6)	10 (28.6)	3 (50.0)	3 (20.0)	ns
Drug abuses, *n* (%)	2 (3.6)	1 (2.9)	1 (16.7)	0 (0.0)	ns
Dialysis, *n* (%)	2 (3.6)	2 (5.7)	0 (0.0)	0 (0.0)	ns
History of PCI, *n* (%)	19 (33.9)	14 (40.0)	1 (16.7)	4 (26.7)	ns
post CPR, *n* (%)	15 (26.8)	9 (25.7)	0 (0.0)	6 (40.0)	ns
Biventricular failure, *n* (%)	31 (55.4)	17 (48.6)	4 (66.7)	10 (66.7)	ns
ACS/ICM, *n* (%)	44 (78.0)	31 (88.6) *	3 (50.0)	10 (66.7)	<0.05
DCM, *n* (%)	8 (14.3)	3 (8.6)	1 (16.7)	4 (26.7)	ns
Myocarditis, *n* (%)	2 (3.6)	1 (2.9)	1 (16.7)	0 (0.0)	ns
CS after oHTX, *n* (%)	2 (3.6)	1 (2.9)	0 (0.0)	1 (6.7)	ns
Postoperative use, *n* (%)	26 (46.4)	17 (48.6)	2 (33.3)	7 (46.7)	ns
va-ECMO implantation, *n* (%)	43 (76.8)	26 (74.3)	6 (100.0)	11 (73.3)	ns
Pulmonary edema, *n* (%)	36 (64.3)	22 (62.9)	5 (83.3)	9 (60.0)	ns
Lactate (mmol/dL)	2.33 ± 1.28	4.47 ± 4.76	1.70 ± 0.86	4.76 ± 4.21	ns
Creatinine (mg/dL)	1.69 ± 0.65	1.69 ± 0.91	1.45 ± 0.54	1.63 ± 0.96	ns
Bilirubin (mg/dL)	5.17 ± 4.21	2.70 ± 2.86	4.56 ± 4.60	2.39 ± 2.72	ns
CRP (mg/dL)	15.8 ± 12.9	12.8 ± 7.80 *	17.7 ± 11.8	7.95 ± 8.27	<0.05
Mortality, *n* (%)	33 (57.9)	20 (57.1)	2 (33.3)	11 (73.3)	ns

Data documented as *n* (%) or mean ± standard deviation. ACS, acute coronary syndrome; COPD, chronic obstructive pulmonary disease; CPR, cardiopulmonary resuscitation; CRP, C-reactive protein; CS, cardiogenic shock; DCM, dilated cardiomyopathy; HIT, heparin-induced thrombocytopenia; ICM, ischemic cardiomyopathy; INTERMACS, interagency registry for mechanically assisted circulatory support; ns, not significant; oHTX, orthotopic heart transplantation; PCI, percutaneous coronary intervention; va-ECMO, venous-arterial extracorporeal membrane oxygenation; *, statistical significance.

**Table 2:** Platelet counts decrease due to HIT.

	Platelets		
	Maximum (* 1000/µL)	Minimum (* 1000/µL)	Rate of Decrease (%)
Case 1	191	25	86.9
Case 2	258	38	85.3
Case 3	100	35	65.0
Case 4	194	33	83.0
Case 5	193	37	80.8
Case 6	167	74	55.7
average			76.1

HIT, heparin-induced thrombocytopenia. * multiplication

**Table 3:** HIT-associated clinical features and outcomes.

	HIT Positive (*n* = 6)	HIT Negative (*n* = 15)	*p*
HIT test after first heparin (days)	10.5 ± 2.89	8.00 ± 4.90	0.15
Event for the first heparin administration since admission			
va-ECMO implantation, *n* (%)	3 (50.0)	7 (46.7)	1.0
Open heart operation, *n* (%)	3 (50.0)	7 (46.7)	1.0
Impella, *n* (%)	0 (0.0)	1 (6.67)	1.0
HIT-associated thrombotic events under Impella support			
Impella dysfunction, *n* (%)	1 (16.7)	-	-
Left ventricular thrombus, *n* (%)	1 (16.7)	-	-

Data documented as *n* (%) or mean ± standard deviation. HIT, heparin-induced thrombocytopenia; va-ECMO, venous-arterial extracorporeal membrane oxygenation

**Table 4:** Clinical characteristic of 4 survival patients with HIT under Impella 5.0 support.

	Patient 1	Patient 2	Patient 3	Patient 4
Case Nr. in Table 2.	Case 2	Case 3	Case 4	Case 5
Basis diagnosis	ACS/ICM	ACS/ICM	myocarditis	DCM
Postcardiotomy?	Yes (CABG)	No	No	No
Impella size	5.0	5.0	5.0	CP → 5.0
va-ECMO?	Yes	Yes	Yes	Yes
Impella duration at HIT positive (d)	1	4	10	11
Successful Impella weaning?	Yes	Yes	Yes	Transition to oHTX
Total Impella duration (d)	7	27	10	12
Coagulopathy?	Yes	Yes	No	No
Systemic anticoagulation				
Before-HIT diagnostic	Heparin	None	Heparin	Heparin
Post-HIT diagnostic	Argatroban	None	Argatroban	Argatroban
Purge anticoagulation				
Before-HIT diagnostic	Heparin 20 U/mL	Heparin 50 U/mL	Heparin 50 U/mL	Heparin 50 U/mL
Post-HIT diagnostic	Argatroban 25 mg/L	Argatroban 20 mg/L	Argatroban 90 mg/L	Argatroban 40 mg/L
Neurological complications?	No	No	No	No
Discharge (d)	55	63	27	44

ACS, acute coronary syndrome; CABG, coronary artery bypass grafting; d, days; DCM, dilatative cardiomyopathy; HIT, heparin-induced thrombocytopenia; ICM, ischemic cardiomyopathy; Nr., number; oHTX, orthotopic heart transplantation; U, units; va-ECMO, venous-arterial extracorporeal membrane oxygenation.

## Data Availability

The data that support the findings of this study are available from the corresponding author upon reasonable request.

## Supplementary Materials

The following are available online at https://www.mdpi.com/article/10.3390/jcdd8120161/s1, Table S1: Clinical course in case 4; Impella dysfunction due to pump thrombosis associated with HIT, Table S2: Clinical course in case 5; left ventricular thrombus associated with HIT under Impella 5.0 support.
